# Reliability of a new French-language triage algorithm for out-of-hours primary care calls: the SALOMON rule

**DOI:** 10.1186/cc14486

**Published:** 2015-03-16

**Authors:** E Brasseur, A Ghuysen, AF Donneau, V D'Orio

**Affiliations:** 1 C.H.U. of Liège, Belgium; 2 University of Liège, Belgium

## Introduction

Because of increased workloads associated with primary care physician (PCP) shortage, many western countries have been facing the difficult challenge of optimizing their out-of-hours primary care services. PCPs initially gathered in small rotation groups, and then further collaborations led to larger-scale cooperatives. In such models, implementation of patient call triage is mandatory to increase the efficiency, quality and safety of care [1]. Organization models differ, from the PCP performing all patient calls to nurses and nurse assistants answering calls and performing triage, but no validated triage algorithm has been reported to date. We developed a specific French-language triage algorithm called SALOMON (Système Algorithmique Liégeois d'Orientation pour la Médecine Omnipraticienne Nocturne) in order to guide nurse triage PCP out-of-hours calls.

The present study tested this algorithm reliability.

## Methods

The SALOMON algorithm is based on 53 common presentation flowcharts using specific discriminators to triage calls into four categories according to the level of care required: emergency medical services, nonemergent visit to local emergency department, PCP home visit or PCP delayed consultation. Using an appropriate statistical test, we assessed the accuracy of presentation flowchart and triage category selections attributed to 130 clinical scenarios, by 10 different nurses, in comparison with references established by a local team of experts, at two different time periods: immediately after training (T0) and 3 to 6 months after algorithm practice (T1).

## Results

Overall selection of flowcharts was accurate for 94.1% of scenarios at T0 and 98.7% at T1. Triage category selection was correct for 93.3% of scenarios at T0 and 98.4% at T1. Both flowchart selection and triage category were correct in of 89.5% case in T0 and at 97.5% T1. When an incorrect flowchart was used, triage category remained accurate in 64.9% and 70.5% respectively. Both flowchart and triage selection accuracy improved significantly from T0 to T1 (*P *< 0.0001).

## Conclusion

The Results of the present study revealed that using the SALOMON algorithm is reliable for out-of-hours PCP call triage by nurses. Validity of this rule may be further evaluated through its actual implementation in real-life conditions.
